# Dietary patterns, untargeted metabolite profiles and their association with colorectal cancer risk

**DOI:** 10.1038/s41598-023-50567-6

**Published:** 2024-01-26

**Authors:** Stina Bodén, Rui Zheng, Anton Ribbenstedt, Rikard Landberg, Sophia Harlid, Linda Vidman, Marc J. Gunter, Anna Winkvist, Ingegerd Johansson, Bethany Van Guelpen, Carl Brunius

**Affiliations:** 1 Department of Diagnostics and Intervention, Oncology, Umeå University, Umeå, Sweden; 2 Department of Clinical Sciences, Pediatrics, Umeå University, Umeå, Sweden; 3 Department of Surgical Sciences, The EpiHub, Uppsala University, Uppsala, Sweden; 4 Department of Life Sciences, Chalmers University of Technology, Gothenburg, Sweden; 5 International Agency for Research On Cancer, Nutrition and Metabolism Section, 69372 Lyon Cedex 08, France; 6 Cancer Epidemiology and Prevention Research Unit, Department of Epidemiology and Biostatistics, School of Public Health, Imperial College London, London, UK; 7 Department of Internal Medicine and Clinical Nutrition, Institute of Medicine, Sahlgrenska Academy, University of Gothenburg, Gothenburg, Sweden; 8 Sustainable Health, Department of Public Health and Clinical Medicine, Umeå University, Umeå, Sweden; 9 Department of Odontology, Section of Cariology, Umeå University, Umeå, Sweden; 10 Wallenberg Centre for Molecular Medicine, Umeå University, Umeå, Sweden

**Keywords:** Risk factors, Colorectal cancer, Biomarkers

## Abstract

We investigated data-driven and hypothesis-driven dietary patterns and their association to plasma metabolite profiles and subsequent colorectal cancer (CRC) risk in 680 CRC cases and individually matched controls. Dietary patterns were identified from combined exploratory/confirmatory factor analysis. We assessed association to LC–MS metabolic profiles by random forest regression and to CRC risk by multivariable conditional logistic regression. Principal component analysis was used on metabolite features selected to reflect dietary exposures. Component scores were associated to CRC risk and dietary exposures using partial Spearman correlation. We identified 12 data-driven dietary patterns, of which a *breakfast food* pattern showed an inverse association with CRC risk (OR per standard deviation increase 0.89, 95% CI 0.80–1.00, p = 0.04). This pattern was also inversely associated with risk of distal colon cancer (0.75, 0.61–0.96, p = 0.01) and was more pronounced in women (0.69, 0.49–0.96, p = 0.03). Associations between *meat*, *fast-food, fruit soup/rice* patterns and CRC risk were modified by tumor location in women. Alcohol as well as fruit and vegetables associated with metabolite profiles (Q^2^ 0.22 and 0.26, respectively). One metabolite reflecting alcohol intake associated with increased CRC risk, whereas three metabolites reflecting fiber, wholegrain, and fruit and vegetables associated with decreased CRC risk.

## Introduction

Diet is considered to play a major role in colorectal cancer (CRC) development, demonstrated mainly in observational studies but also in randomized control trials^1^. Red and processed meat^2^ and alcohol^2,3^ have been convincingly associated with increased risk of CRC^4^. For dietary fiber^5^, whole grains^2^, dairy foods^2,6^, and calcium intake (supplementary and dietary in the form of dairy products)^7^, a probable decreased risk has been suggested^2,4,8^. However, even for some of the best-established dietary risk- and protective factors, results have not been entirely consistent^9,10^ and non-linear relationships have been suggested^11^. The difficulty in identifying dietary components with a clear impact on CRC risk has led to novel approaches acknowledging the complexity of diet. One such approach is dietary pattern analysis, which may provide more accurate measurements of dietary exposure by taking complex interactions into account^12,13^.

Dietary pattern analyses have predominantly been based on a priori hypotheses for the role of individual dietary components in health and disease, such as the Mediterranean diet score^14^, which we have previously investigated together with the Dietary Inflammatory Index^15^ but with no found associations to CRC risk in a large longitudinal cohort study with repeated measures^16^. Hypothesis-driven dietary patterns applied to CRC, including putative risk and protective factors, have shown associations with risk in some studies^17–20^, but not all^8,20^. However, most patterns used have not been designed specifically for CRC, and components of particular interest for CRC, such as dairy products and alcohol, are scored beneficial in some patterns and detrimental in others^14,16,21^. These discrepancies, which likely stem from differences in the populations, food cultures, possible biological mechanisms and disease outcomes on which they are based^12^, illustrate the need for clear definitions but also additional methods to address the complex role of diet in CRC development. Another dietary pattern-based approach which may contribute with additional information is to derive data-driven patterns, based directly on intake frequency data from the population under study. Principal component analysis (PCA) and factor analysis are methods that can be used to reduce the dimensionality of multiple dietary predictors into underlying latent variables, corresponding to a posteriori (data-driven) dietary patterns^12,22^. For CRC risk, findings from the few studies conducted to date have been inconclusive^23–26^. To distinguish between the two approaches, we consistently refer to hypothesis-driven dietary components and data-driven dietary patterns throughout the paper.

The comprehensive assessment of metabolites in blood samples, i.e. metabolomics, presents opportunities to study metabolic perturbations in relation to phenotype, which can contribute to a better understanding of the etiology of diet-related diseases like CRC^27^. When exposed to diet, individuals are also exposed to the food metabolome, which entails thousands of bioactive food constituents^28^. All of these may affect metabolism and, subsequently, the endogenous metabolome. The food metabolome may therefore represent a potential source of biomarkers for the diet, unencumbered by the bias and noise of self-reported dietary data typically used in epidemiological studies^29,30^.

Here, we investigated data-driven dietary patterns and other dietary components, selected a priori based on putative effects on CRC risk, in relation to subsequent CRC risk in 776 prospective CRC cases and 776 control participants from the population-based Northern Sweden Health and Disease Study. Using prospectively collected pre-diagnostic plasma samples from 680 of these matched case–control pairs, we also explored associations of dietary patterns and a priori dietary components with the plasma metabolome measured by untargeted reverse phase liquid chromatography–mass spectrometry (LC–MS).

## Methods

### Study population

We used data and plasma samples from the population-based cohorts of the Northern Sweden Health and Disease Study (NSHDS). Participants in this study were recruited between 1991 and 2014 to either the Västerbotten Intervention Programme (VIP) (91.3%) or the Northern Sweden MONICA (Multinational Monitoring of Trends and Determinants in Cardiovascular Disease) study (8.7%), which have been described in detail previously^31,32^. In brief, the VIP is an ongoing screening and intervention program for cardiometabolic health. It has been running in the county of Västerbotten in northern Sweden since 1986. People turning 40, 50, and 60 years are invited, with a full population intent, to a voluntary health examination at their local healthcare center, at which fasting blood samples are taken, an oral glucose tolerance test is administered, and extensive questionnaires, including FFQs, are collected. Anthropometric measurements are taken, as well as blood pressure and cholesterol levels. Participation rates have generally been high, roughly 50–70% of the target population in Västerbotten^33^. In the MONICA cohort, participants aged 25–74 years are randomly recruited from the counties of Västerbotten and Norrbotten approximately every 5 years^31^. Sampling for the MONICA cohort follows nearly identical protocols as the VIP^32^.

### Study design

The present study had a nested case–control study design. Cases of first primary CRC were identified by linkage to the essentially complete Cancer Registry of Northern Sweden. Previous cancer other than non-melanoma skin cancer was an exclusion criterion. Information about histology (only adenocarcinomas were included) and anatomical tumor location was retrieved from the Swedish Colorectal Cancer Registry, supplemented by patient pathology records when necessary. Using ICD-10 codes, tumor site was defined as the proximal colon (C.18.0 and C 18.2–18.4), distal colon (C18.5–18.9), or rectum (C19.9 and C20.9). End of follow-up for identification of cases was May 31, 2016.

Control participants were individually matched to cases by cohort (VIP/MONICA), sex, age at baseline (± 1 year), year of blood sampling and data collection (± 1 year), fasting duration at sample collection (above or below 8 h), and number of freeze–thaw cycles of the plasma samples. Controls had to have no previous cancer diagnosis (other than non-melanoma skin cancer) at the time of the CRC diagnosis of their corresponding case.

The research in this study was approved by the Research Ethics Committee at Umeå University, Umeå, Sweden (Dnr 2015/243-32 and Dnr 2017-172-32). At recruitment, informed consent was collected from all participants. All data handling complies with the European Union General Data Protection Regulation. The study conforms with The Code of Ethics of the World Medical Association (Declaration of Helsinki), printed in the British Medical Journal (18 July 1964).

### Dietary data

Dietary data were retrieved from the Northern Sweden Diet Database, which comprises harmonized dietary data from the validated FFQs collected from participants in the Northern Sweden Health and Disease Study (https://www.umu.se/en/biobank-research-unit/)^34,35^. Participants reported intake frequencies for the previous year covering food consumption for all seasons, weekdays, and weekends, on a 9-level scale from “never” to “≥ 4 times per day”. The frequency data and portion-size estimations of vegetables, typical carbohydrate, and protein sources, based on photographs of four standard portion sizes, were used to calculate amounts using the national food composition database^36^.

Data-driven (a posteriori) dietary patterns were identified using a combination of exploratory and confirmatory factor analysis. Dietary data were represented by the reported frequencies for all items in the FFQ. We considered using amounts, but preliminary analyses yielded very similar results and based on the exploratory nature of this investigation, and for simplicity, we chose frequencies. First, dietary data from all individuals were randomly split into halves. One half was used to identify potential dietary patterns from exploratory factor analysis using maximum likelihood factorization and *oblimin* rotation (the *fa* function from the R package *psych* v 1.9.12.31). The other half was used for confirmatory factor analysis, in which factors were constructed from those dietary variables that had loading > 0.3 (the *cfa* function from the R package *lavaan* v 0.6-6). The exploratory/confirmatory factor analysis random split procedure was repeated 5 times and factors that appeared reproducibly between the repeated half-splits were selected to represent potential constructs and examined further. The repeated exploratory/confirmatory factor analysis procedure was performed for 2–18 factors. A combined assessment of averaged fitness measures from the confirmatory factor analysis indicated similar modelling fitness between 9 and 18 factors. Extracted factors from these solutions were inspected to identify constructs that occurred reproducibly between different numbers of factors, resulting in the identification of 12 data-driven dietary patterns (Table 1). A final confirmatory factor analysis was constructed for these 12 dietary patterns and participant scores were extracted and used for metabolomics analyses and CRC risk assessment. From the exploratory and confirmatory factor analyses, dietary variable loadings were extracted to show direction of intake in relation to the factor scores.

**Table 1:** Foods included in data-driven dietary patterns produced using exploratory and confirmatory factor analysis, and in hypothesis-driven dietary components based on putative etiological roles in colorectal cancer.

	Included foods
*Dietary patterns (a posteriori), based on food frequency data*^*a*^	
Breakfast food	Fermented milk products: Low- and 3%-fat Swedish “filmjölk” and yoghurt, fiber-rich breakfast cereals, berries (fresh and frozen)
Spreads	Butter and margarine on bread
Bread with low-fat spreads	Low-fat margarine and cheese on bread, whole-grain crisp bread, buns,
Full fat products	Butter on bread, butter for cooking, milk with 3% fat
Vegetables	Root vegetables, carrots, tomato, cucumber
Fruit soup and rice	Rosehip syrup/soup, rice
Fish	Lean fish, fatty fish, salty fish
Meat	Minced-meat dishes, meat stew, steak/chops
Smoked	Smoked fish and smoked meat
Fast food	Pizza, hamburger, bacon, sausage
Snacks and sweets	Potato chips, salty nuts, popcorn, buns, sweets, cookies/pastry
Alcohol	Medium strong beer (2.8–3.5%), strong beer (≥ 4.5%), wine, spirit/liquor
*Dietary components (a priori)*^*b*^*, based on food intake in amounts/day*^*c*^	
Dairy products	Crème fraiche, cheese, low- and 3%-fat Swedish “filmjölk” and yoghurt, milk 0,5%, milk, milk 1,5%, Swedish “filmjölk” 1,5%, milk 3%
Dietary calcium	Total intake of dietary calcium from various sources
Wholegrain	Total wholegrain intake from various sources
Fiber	Total fiber intake from various sources
Fruit and vegetables	Berries, fresh or frozen, apple, pear, peach, orange, mandarin, grapefruit, banana, white cabbage, root vegetables & carrots, tomatoes & cucumber, lettuce, lettuce cabbage, spinach, broccoli
Red meat	Minced meat dishes, meat stew, steak chop
Processed meat	Bacon and sausage as main dish, sausage, meat, and liver paté on bread
Red and processed meat	Minced meat dishes, meat stew, steak chop, hamburger, bacon and sausage as main dish, sausage, meat, and liver paté on bread
Total alcohol	Alcohol from light beer (2.1%), medium strong beer (2.8–3.5%), strong beer (4.5%), wine, spirit/liquor

^a^All patterns were produced using food frequency data (e.g., frequency/day) with no information about amounts of intake.

^b^Based on global scientific research on diet, nutrition, physical activity and the risk of colorectal cancer and reported by the World Cancer Research Fund Global Cancer Update Programme^4^.

^c^All components were composed by amount data, recalculated into g/day or mg/day using frequency data and information about portion sizes.

We also assessed a priori hypothesis-driven dietary components: alcohol, red meat, processed meat, wholegrain, fiber, fruit and vegetables, dairy products, and dietary calcium, based on the state of the art with respect to evidence for an etiological role in CRC, summarized in the World Cancer Research Fund’s Global Cancer Update Programme^4^. All dietary patterns and food components were energy adjusted using the energy–density method^37^.

### Baseline covariates

All lifestyle variables were self-reported. Smoking status was defined as either non-smoker, ex-smoker, or current smoker. Recreational physical activity was defined on a scale from 1 to 4 (no, low, medium, high frequency of physical activity). Total alcohol intake was assessed from the FFQ and, when included as a potential confounder in multivariable analyses, defined at three levels: zero intake and intake below or above the sex-specific non-zero median. This categorization took into consideration the potentially mixed group of non-consumers, including both alcohol abstainers and former over-consumers. Education was classified into three levels: elementary school, secondary school, or post-secondary education. Body mass index (BMI, kg/m^2^), was calculated from height and weight data, recorded by medical staff at recruitment and used on the continuous scale.

### Plasma samples

Blood samples were collected at the same time point as the baseline covariate data. The vast majority of the baseline plasma samples, 1238 (91.0%) of the participants in this study, were taken after an overnight fast (> 8 h), 82 samples (6.1%) were taken after 4–8 h fasting, and 40 samples (2.9%) after less than 4 h fasting. The blood samples were collected in EDTA tubes and separated immediately into plasma, buffy coat and erythrocyte fractions. Within 1 h after collection, samples were stored at − 20 °C for a maximum of 1 week before transfer for central storage at – 80 °C at Biobank North in Umeå, Sweden.

### Metabolomics analysis

Metabolomics procedures are described in detail elsewhere^38^. In brief, aliquoted plasma samples ordered to preserve case–control pairs (with random sorting within pairs) were cold-shipped at − 80 °C to the Chalmers Mass Spectrometry Infrastructure at Chalmers University of Technology, Gothenburg, Sweden. Proteins were precipitated using cold acetonitrile in 96-deep well microplates, mixed on an orbital shaker for 3 min at 1000 rpm, centrifuged and filtered. The filtrate was collected in 96-well microplates, centrifuged, and kept at 4 °C until instrumental analysis. Study-specific quality control samples (sQCs) were obtained by pooling sample aliquots from the first two batches and were systematically and repeatedly injected throughout the batch sequence. To correct for batch effects and to monitor the performance of the instrument, independent long-term quality control plasma samples (ltQCs) were used^39^.

The Liquid-Chromatography Mass-spectrometry (LS-MS) analysis was performed on an Agilent UHPLC-qTOF-MS system (1290 UHPLC with a 6550 qTOF). Analytes were separated by reverse phase chromatography on a Waters Acquity UPLC HSS T3 column (100 × 2.1 mm, 1.8 µm). The Agilent MassHunter workstation was used to operate and monitor the instrument and acquire data. The mobile phase included (A) water and (B) methanol, both containing 0.04% formic acid. The linear gradient elution was: 0–6 min, 5–100% B, 6–10.5 min, 100% B, delivered at 0.4 mL/min. Metabolites were ionized by Jetstream electrospray ionization (ESI). The mass spectrometer was operated in both positive and negative modes, with 2 and 4 µL injected for positive and negative modes, respectively. Data were acquired within m/z 50–1600 in centroid mode at 1.67 spectra/s. Iterative MS/MS data acquisition was performed on sQC samples in both modes with 10, 20 and 40 eV collision energies and with the same chromatographic conditions as for the MS analysis.

### Data pre-processing

Vendor raw data files were converted into mzML format, processed separately for reverse phase positive (RP) and negative (RN) modes (Proteo Wizard, version 3.0) and processed using the R package “XCMS”^40^, with key parameters optimized with the aid of the R package “IPO”^41^. A total of 8236 metabolite features were obtained for RP and 6599 features for RN. Imputation for missing values in the metabolomics data was conducted using an in-house random forest (RF) based algorithm (https://gitlab.com/CarlBrunius/StatTools). Within- and between-batch normalization were performed using R package “BatchCorr”^39^. Finally, features presumably derived from the same metabolite were grouped with the R package “RAMClustR”^42^ using manually optimized parameters, which resulted in 2644 features for RP and 2391 features for RN with coefficient of variation (CV) ≤ 30% among sQCs. Parameters used are presented in Suppl. Table  1.

### Metabolite identification

Metabolite identification was carried out using an in-house native standard library and the Massbank of North America^43^, as well as the in-silico fragmentation tools MetFrag^44^ and Sirius^45^. All files containing MS2 spectra were converted to mgf format prior to analysis. Identification was carried out according to the Schymanski scale, determining the confidence level (CL) on a scale from 1 to 5^46^. For library comparisons, a modified cosine score above 0.9 was determined as a CL 1 match. Any feature which obtained an exact spectral similarity score above 0.9 in MetFrag was determined as a CL 2 match. For features of which most spectra were predicted to be the same compound by both MetFrag and Sirius, a CL 3 was assigned. When most of the spectra of a feature were predicted to be the same compound in either MetFrag or Sirius, but not by both, a CL 3–4 was assigned, depending on manual assessment of spectral similarity. When most of the spectra were predicted to have the same chemical formula in Sirius, a CL 4 was assigned and when no MS2 was obtained for a feature, or when there was no majority of spectral predictions, the feature was assigned a CL 5. Parameters for Sirius, MetFrag, HMDB, and in-house library matching are found in Suppl Table 1.

### Statistical analysis

For all data- and hypothesis-driven dietary patterns and components, we investigated the association with CRC risk by multivariable conditional logistic regression, adjusted for BMI, smoking, physical activity, education, total energy intake, and alcohol. Association between diet and CRC risk were evaluated by estimating odds ratios (ORs) per 1 standard deviation (SD) increase in frequency/day or gram/day in dietary pattern or dietary component, respectively. When alcohol was the main exposure (used as a continuous variable), alcohol (categorized) was not included as a covariate. The analyses were considered exploratory, and the significance threshold was consequently set at nominal P < 0.05. The few participants with missing values for some covariates (presented in Table 2) were omitted in the statistical analyses. In addition, stratified analyses were performed by sex (women and men), and by tumor location (proximal colon, distal colon, and rectum).

**Table 2:** Baseline characteristics of 680 prospective colorectal cancer cases and 680 matched controls with complete dietary and metabolomics data.

Variable	Total, n = 1360	Cases, n = 680	Controls, n = 680
Age at baseline, years, median (IQR)	59.7 (50.0–60.0)	59.7 (49.9–60.0)	59.7 (50.0–60.0)
Follow-up time, years, median (IQR)	11.3 (6.4–15.6)	11.3 (6.4–15.5)	11.3 (6.5–15.7)
Sex, n (%)			
Men	688 (50.6)	344 (50.6)	344 (50.6)
Women	672 (49.4)	336 (49.4)	336 (49.4)
Cohort, n (%)			
VIP	1242 (91.3)	621 (91.3)	621 (91.3)
MONICA	118 (8.7)	59 (8.7)	59 (8.7)
BMI kg/m^2^, n (%)			
< 25 normal weight	527 (38.8)	248 (36.5)	279 (41.0)
25–30 overweight	606 (44.6)	311 (45.7)	295 (43.4)
> 30 obese	219 (16.1)	117 (17.2)	102 (15.0)
Missing	8 (0.4)	4 (0.4)	4 (0.74)
BMI kg/m^2^, mean (sd)	26.4 (4.0)	26.6 (4.1)	26.2 (3.8)
Smoking status, n (%)			
Never smoker	572 (42.1)	272 (40.0)	300 (44.1)
Ex-smoker	476 (35.0)	248 (36.5)	228 (33.5)
Current smoker	298 (21.9)	151 (22.2)	147 (21.6)
Missing	14 (1.0)	9 (1.3)	5 (0.7)
Recreational physical activity level, n (%)			
None	575 (42.4)	298 (43.9)	277 (40.9)
Low (occasionally)	347 (25.6)	172 (25.3)	175 (25.8)
Medium (1–3 times/w)	356 (26.2)	175 (25.8)	181 (26.7)
High (> 3 times/w with higher intensity)	65 (4.8)	29 (4.3)	36 (5.3)
Missing	17 (1.3)	6 (0.9)	11 (1.6)
Educational level, n (%)			
Elementary school	511 (37.6)	245 (36.0)	266 (39.1)
Secondary school	600 (44.1)	315 (46.3)	285 (41.9)
Post-secondary school	240 (17.6)	115 (16.9)	125 (18.4)
Missing	9 (0.7)	5 (0.7)	4 (0.6)
Civil status, n (%)			
Unmarried	101 (7.4)	44 (6.5)	57 (8.4)
Married or cohabitant	1091 (80.2)	546 (80.3)	545 (80.1)
Separated	98 (7.2)	46 (6.8)	52 (7.6)
Widow/widower	53 (3.9)	37 (5.4)	16 (2.4)
Missing	17 (1.3)	7 (1.0)	10 (1.5)
Alcohol intake, g/day, n (%)			
Zero intake	121 (8.9)	57 (8.4)	64 (9.4)
Below median (sex-specific)	559 (41.1)	295 (43.4)	264 (38.8)
Above median (sex-specific)	680 (50.0)	328 (48.2)	352 (51.8)
Alcohol intake, g/day, mean (sd)	4.0 (4.8)	4.1 (5.1)	3.9 (4.4)
Energy intake, kcal/day, mean (sd)	1704 (637)	1683 (644)	1724 (29)

*IQR* interquartile range, *BMI* body mass index, *VIP* Västerbotten Intervention Programme, *MONICA* Multinational Monitoring of Trends and Determinants in Cardiovascular Disease.

Associations between dietary exposures factors and metabolome were investigated using random forest regression. Metabolomics data were entered as explanatory variables and each of the energy-adjusted data-driven patterns, underlying dietary variables, as well as a priori components were entered as response variables. The data were processed using the R MUVR package v 0.0.973, which employs a repeated double cross-validation framework incorporated with unbiased variable selection^47^. In addition, samples used for predictions in such nested cross-validation are never used for either parameter tuning, or model training and predictions are therefore not subject to overfitting. Models considered potentially informative at predictive performance (Q^2^) > 0.15 were further assessed by permutation analysis (n = 50) to assess modelling performance^48^. Metabolite features selected from MUVR models with Q^2^ > 0.15 and P_permutation_ < 0.05, were further validated using partial Spearman correlation with their corresponding dietary exposure at the baseline measurement while adjusting for the same covariates as in the conditional logistic regression models.

Associations of individual metabolite features selected to reflect dietary exposures (n = 36) with CRC risk was performed as above. In addition, associations between dietary metabolite profile, exposures and CRC risk were investigated using our in-house R-based ‘triPlot’ algorithm (https://gitlab.com/CarlBrunius/triplot)^49^. In brief, a principal component analysis (PCA) was performed on the set of metabolite features selected to reflect dietary exposures (n = 36) for all case–control pairs at baseline that had complete data, including metabolite features (n = 680). Component scores were associated to CRC risk as above and to dietary exposures using partial Spearman correlation, adjusted for the same potential confounders. Associations were visualized in a triplot with the metabolite loadings from the PCA, superimposed with exposure correlations and ORs for risk of developing CRC.

All statistical analyses were conducted in the statistical software R v 4.0.2 (Foundation for Statistical Computing, Vienna, Austria). Baseline descriptions were performed in IBM SPSS statistics, version 28.

## Results

### Study participants

**Figure 1:**
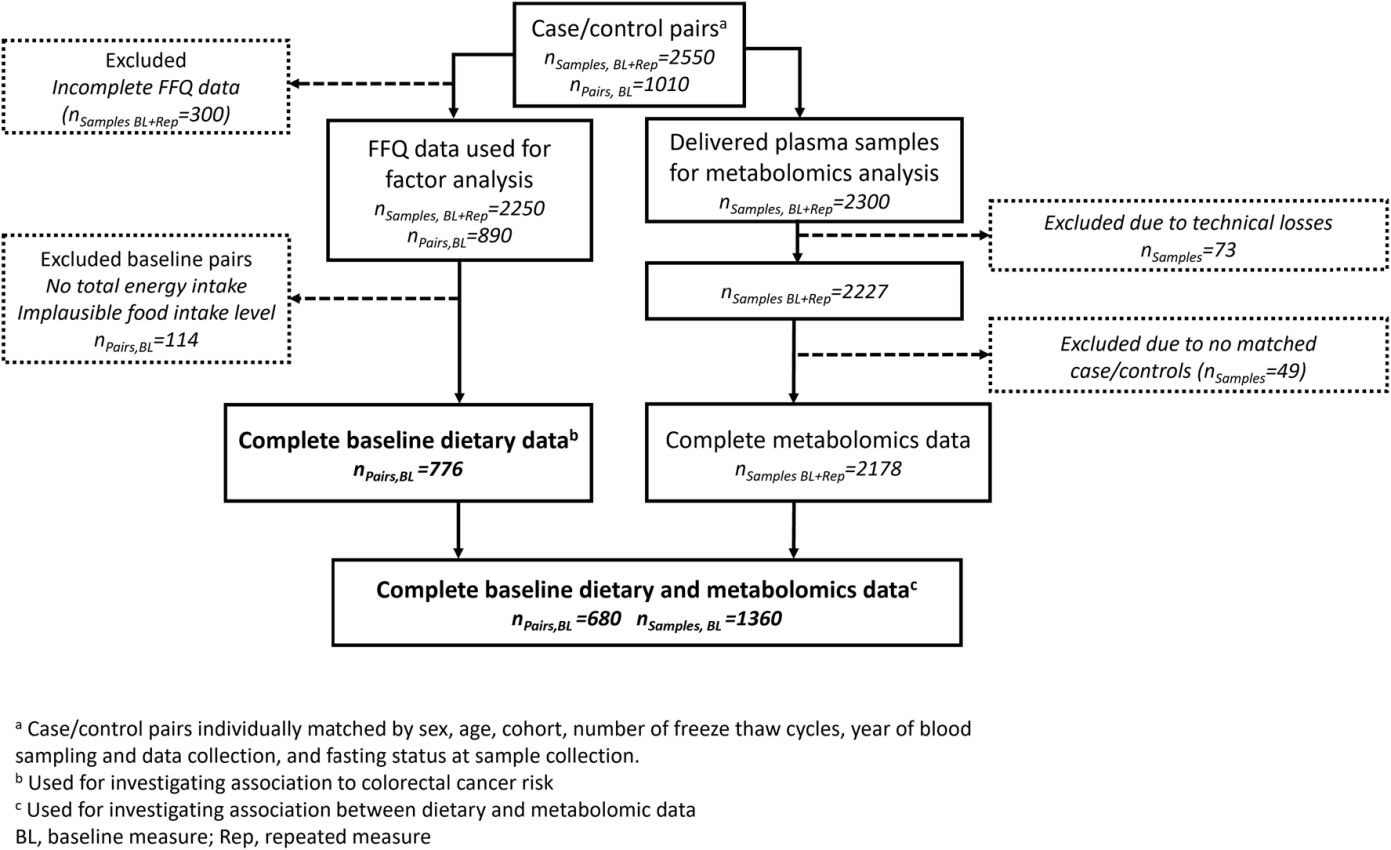
Flow chart. Inclusion and exclusion of study participants from the Northern Sweden Health and Disease Study with baseline sampling of plasma and dietary data from March 1991 to April 2014 and a median follow-up time from baseline to the CRC diagnosis cases of 11.3 years.

In total, 2550 samples, including baseline (BL) and repeated (Rep) samples from 1010 case–control pairs, were originally obtained (Fig. 1). All samples were collected prior to the CRC diagnosis of the case in each case–control pair. After exclusions for incomplete FFQ data, 2250 samples from 890 pairs were used to produce dietary patterns and 776 pairs were analyzed for diet-CRC association. After further exclusions for insufficient metabolomics data, 680 incident CRC cases and 680 matched controls, recruited 1991–2014 were available for all analyses. Of the 680 matched pairs, 621 (91.3%) were from the VIP cohort, and 59 (8.7%) were from the MONICA cohort. Three CRC cases had unknown tumor site and were thus removed from the site-specific analyses. For participants with more than one health examination or blood sample available in the final data set, the first occasion was used in the analyses. Characteristics of the participants from the baseline measurement are presented in Table 2. Median follow-up time from baseline to CRC diagnosis was 11.3 years. Compared to participants excluded from the study, included participants were older (more often recruited at 60 than 50 years of age), had somewhat shorter follow-up times and had a higher proportion of women. (Suppl. Table 2).

### Data-driven dietary patterns and risk of colorectal cancer

From the combination of exploratory and confirmatory factor analysis, we identified 12 robust dietary patterns named: *breakfast food, low-fat, smoked, fruit soup and rice, snacks and sweets, spreads, vegetables, meat, full fat, fish, fast food,* and *alcohol pattern*. The constituents of each pattern are presented in Table 1, together with a list of the dietary components selected a priori*,* based on their putative role in CRC risk. Dietary variable loadings showing the direction of intake in relation to the factor scores produced by the exploratory and confirmatory factor analyses can be found in Suppl. Table 3.

From the multivariable conditional logistic regression model, the *breakfast food* pattern showed lower overall CRC risk (OR per 1 standard deviation increase 0.89, 95% CI 0.80–0.997 P = 0.04) (Table 3). The *breakfast food* pattern was inversely associated with the risk of distal colon cancer (OR 0.75, 95% CI 0.61–0.96, P = 0.01), but not proximal colon cancer (OR 1.04, 95% CI 0.84–1.29, P = 0.69) or rectal cancer (OR 0.88, 95% CI 0.73–1.07, P = 0.20) (Table 4). Furthermore, the association between the *breakfast food* pattern and distal colon cancer risk was more pronounced in women (OR 0.69, 95% CI 0.49–0.96, P = 0.03) than in men (OR 0.79, 95% CI 0.58–1.08, P = 0.13) (Table 4). A pattern with connection to breakfast food, containing fruit soup and rice meals, was also inversely associated with distal colon cancer in women (OR 0.64, 95% CI 0.43–0.95, P = 0.03). Of the dietary components selected a priori, several were represented in the *breakfast food* pattern, i.e., dairy products and fiber-rich breakfast cereals contains dietary calcium, wholegrain, and fiber. There was an inverse association for total dietary calcium intake (OR 0.88, 95% CI 0.79–0.97, P = 0.01) and dairy foods (OR 0.90, 95% CI 0.81–1.00, P = 0.05) and a non-significant inverse association for wholegrain (OR 0.93 95% CI 0.83–1.04, P = 0.22) in relation to CRC risk (Table 3), whereas for dietary fiber the association was null (OR 1.00, 95% CI 0.89–1.11, P 0.95).

**Table 3:** Associations for data-driven dietary patterns and hypothesis-driven dietary components in relation to colorectal cancer (CRC) risk and untargeted plasma metabolite profiles in all study participants, women and men in matched case–control pairs.

	CRC risk association all, n = 1532^a^		Metabolite profile association n = 1360		CRC risk association in women, n = 754		CRC risk association in men, n = 778	
	OR (95% CI)	P	Q^2^	P_permutation_^e^	OR (95% CI)	P	OR (95% CI)	P
*Data-driven dietary patterns, produced by frequency data*^*b,c*^								
Breakfast food	0.89 (0.80–0.997)	0.04	0.00	NC	0.89 (0.75–1.04)	0.14	0.89 (0.76–1.05)	0.16
Smoked	0.96 (0.85–1.09)	0.55	-0.03	NC	0.98 (0.82–1.17)	0.81	0.94 (0.78–1.13)	0.51
Bread with low-fat spreads	0.97 (0.86–1.10)	0.63	0.06	NC	0.91 (0.76–1.09)	0.31	1.03 (0.87–1.22)	0.76
Fruit soup and rice	0.98 (0.88–1.10)	0.77	-0.03	NC	0.93 (0.79–1.09)	0.36	1.05 (0.88–1.25)	0.58
Vegetables	1.00 (0.89–1.12)	0.96	0.08	NC	1.02 (0.87–1.20)	0.81	0.98 (0.82–1.17)	0.83
Snacks and sweets	1.00 (0.88–1.13)	0.99	0.02	NC	1.12 (0.93–1.34)	0.23	0.90 (0.75–1.08)	0.27
Spreads	1.01 (0.90–1.12)	0.93	0.11	NC	1.10 (0.94–1.28)	0.25	0.93 (0.79–1.09)	0.35
Full fat	1.03 (0.92–1.15)	0.64	0.07	NC	1.11 (0.94–1.31)	0.22	0.93 (0.80–1.09)	0.40
Meat	1.04 (0.92–1.17)	0.55	-0.02	NC	0.97 (0.82–1.15)	0.74	1.08 (0.930–1.28)	0.41
Fast food	1.06 (0.93–1.20)	0.38	0.10	NC	1.16 (0.96–1.39)	0.12	0.96 (0.80–1.14)	0.61
Fish	1.08 (0.96–1.21)	0.21	0.06	NC	1.04 (0.88–1.22)	0.67	1.11 (0.95–1.31)	0.19
Alcohol^d^	1.09 (0.97–1.21)	0.13	0.22	< 2.2 * 10^–16^	1.05 (0.89–1.23)	0.59	1.13 (0.97–1.32)	0.12
*Hypothesis-driven dietary components, by amount data (mg or g/day)*^*b,c*^								
Dietary calcium	0.88 (0.79–0.97)	0.01	0.12	NC	0.83 (0.71–0.97)	0.02	0.93 (0.80–1.08)	0.34
Dairy foods	0.90 (0.81–0.997)	0.050	0.11	NC	0.87 (0.75–1.01)	0.08	0.93 (0.81–1.08)	0.37
Wholegrain	0.93 (0.83–1.04)	0.22	0.18	< 2.2 * 10^–16^	0.82 (0.69–0.97)	0.02	1.03 (0.89–1.21)	0.68
Fiber	1.00 (0.89–1.11)	0.95	0.19	< 2.2 * 10^–16^	0.96 (0.81–1.13)	0.61	1.04 (0.89–1.22)	0.61
Fruit and vegetables	1.02 (0.92–1.14)	0.69	0.26	< 2.2 * 10^–16^	1.07 (0.91–1.25)	0.40	1.01 (0.86–1.18)	0.88
Red meat	1.03 (0.92–1.14)	0.64	0.03	NC	0.99 (0.85–1.16)	0.93	1.04 (0.89–1.21)	0.61
Processed meat	1.04 (0.94–1.16)	0.43	0.03	NC	1.10 (0.94–1.28)	0.22	1.01 (0.87–1.18)	0.86
Red and processed meat	1.04 (0.93–1.15)	0.49	0.06	NC	1.03 (0.88–1.20)	0.69	1.04 (0.89–1.21)	0.61
Total alcohol^d^	1.04 (0.94–1.16)	0.43	0.23	< 2.2 * 10^–16^	0.97 (0.83–1.13)	0.68	1.13 (0.97–1.32)	0.11

*CRC* colorectal cancer, *OR* odds ratio, *CI* confidence interval, NC: not calculated.

^a^After exclusion of matched pairs due to missing values in covariates (n_pairs_ = 10).

^b^All dietary patterns and components were energy adjusted using the energy–density method.

^c^Adjusted for potential confounders; BMI kg/m^2^, smoking (never-/ex-/current smoker), physical activity (no/low/medium/high), education (elementary school/secondary school /post-secondary school), total energy intake, kcal/day, and alcohol (non-consumers/below sex-specific median/above sex-specific median intake).

^d^Adjusted for the same potential confounders as for all other dietary exposures, except alcohol.

^e^ Not calculated (NC) if predictive performance (Q^2^) < 0.15.

**Table 4:** Associations for data-driven dietary patterns showing sex- and tumor-site-specific associations with colorectal cancer risk in all participants, women and men in matched case–control pairs.

Dietary patterns^a,b^	All			Women			Men		
	n	OR (95% CI)	P	n	OR (95% CI)	P	n	OR (95% CI)	P
Breakfast food									
Proximal colon	454	1.04 (0.84–1.29)	0.69	272	0.97 (0.73–1.29)	0.82	182	1.25 (0.86–1.81)	0.24
Distal colon	460	0.75 (0.61–0.96)	0.01	210	0.69 (0.49–0.96)	0.03	250	0.79 (0.58–1.08)	0.13
Rectum	612	0.88 (0.73–1.07)	0.20	270	0.93 (0.68–1.25)	0.61	342	0.84 (0.65–1.09)	0.19
Fruit soup and rice									
Proximal colon	454	0.91 (0.74–1.13)	0.39	272	0.99 (0.77–1.28)	0.95	182	0.77 (0.47–1.25)	0.29
Distal colon	460	0.90 (0.72–1.12)	0.36	210	0.64 (0.43–0.95)	0.03	250	1.24 (0.88–1.74)	0.22
Rectum	612	1.10 (0.91–1.32)	0.32	270	1.10 (0.83–1.44)	0.51	342	1.13 (0.86–1.49)	0.37
Meat									
Proximal colon	454	1.00 (0.78–1.29)	0.99	272	0.73 (0.51–1.06)	0.10	182	1.60 (0.98–2.60)	0.06
Distal colon	460	0.90 (0.73–1.12)	0.36	210	0.85 (0.62–1.16)	0.31	250	0.91 (0.65–1.28)	0.60
Rectum	612	1.15 (0.95–1.39)	0.15	270	1.38 (1.00–1.92)	0.05	342	1.06 (0.83–1.36)	0.64
Fast food									
Proximal colon	454	1.03 (0.81–1.32)	0.80	272	0.95 (0.68–1.34)	0.78	182	1.25 (0.86–1.81)	0.24
Distal colon	460	1.02 (0.81–1.28)	0.86	210	1.13 (0.81–1.59)	0.47	250	0.89 (0.63–1.26)	0.52
Rectum	612	1.08 (0.88–1.32)	0.45	270	1.49 (1.04–2.12)	0.03	342	0.92 (0.71–1.20)	0.54

OR: odds ratio; CI: confidence interval.

Estimates for the other eight data-driven patterns are presented in Supplementary Table 2.

^a^Energy adjusted using the energy–density method.

^b^Adjusted for potential confounders; BMI kg/m^2^, smoking (never-/ex-/current smoker), physical activity (no/low/medium/high), education (elementary school/secondary school /post-secondary school), total energy intake, kcal/day, and alcohol (non-consumers/below sex-specific median/above sex-specific median intake).

The *alcohol* pattern consisted of beer with a moderate alcohol content (2.8–3.5%), strong beer (≥ 4.5% alcohol), wine and liquor, and was not significantly associated to overall CRC risk (OR 1.09, 95% CI 0.97–1.21, P = 0.13) (Table 3).

The *meat* pattern, consisting of minced meat dishes, meat stew, steak, and chops, did not associate with overall CRC risk in men or women. However, when stratifying for sex and tumor site, the *meat* pattern associated with increased risk of rectal cancer in women (OR 1.38, 95% CI 1.00–1.92, P = 0.05), (Table 4). The pattern dubbed *fast food*, including pizza, hamburger, bacon, and sausage, was not associated with overall CRC risk but was, like the *meat* pattern, associated with a higher risk of rectal cancer in women (OR 1.49, 95% CI 1.04–2.12, P = 0.03), whereas no such association was found in men (Table 4). Red and/or processed meat intake in g/day did not associate with either overall, sex-, or site-specific CRC risk (Tables 3 and 4).

None of the other eight data-driven dietary patterns were associated with the risk of CRC, either overall (Table 3), or in subgroups stratified by sex (Table 3), or sex and tumor location (Suppl. Table 4).

### Dietary patterns and association to plasma metabolites

Of the analyzed dietary data, including both hypothesis- and data-driven dietary components and patterns, five associated to the metabolome (Table 3). After adjustment for potential confounders (the same potential confounders included in the conditional logistic regression: BMI, smoking, physical activity, education, total energy intake, and alcohol, between 5 and 15 metabolite features correlated with respective dietary exposure variables (Suppl. Table 5).

There was substantial overlap in selected features for the alcohol pattern and total alcohol intake as well as between total intake of wholegrain and dietary fiber, resulting in 36 unique features associated with dietary exposures. Among those, 3 metabolite features measured in negative polarity reflected alcohol intake and associated with increased CRC risk. Two of these features (m/z 224.0623 and 224.5639) co-eluted at 311 s and differed in molecular weight by 0.5 Da, indicating isotopes of a doubly charged, albeit unidentified, metabolite. Interestingly, the third feature had the same m/z ratio and isotope pattern (not shown), but eluted approx. 30 s earlier, partly overlapping with the distinct peak at 310 s, suggesting it to be a structural isomer. This notion was strengthened by high correlations (r ≥ 0.77) and similar associations to alcohol exposure and CRC risk (Suppl. Table  5).

In addition, 3 features associated with decreased CRC risk: One of these features (negative polarity, 91.63 s, m/z 188.0024) reflected both wholegrain and fiber (OR = 0.82 (0.72–0.93), p = 0.0017) and was tentatively annotates as aminophenol sulphate (Level 3). Another feature (unidentified) also reflected fiber intake (positive polarity, 268.53 s, m/z 130.0650 with additional fragments at 131.0685 (isotope), 189.0785 and 190.0859; OR = 0.88 (0.78–1.00), p = 0.042). The third feature (negative polarity, 52.53 s, m/z 129.0206) reflected total intake of fruit and vegetables and was annotated by molecular formula as [C5H6O4-H]^−^ (Level 4).

**Figure 2:**
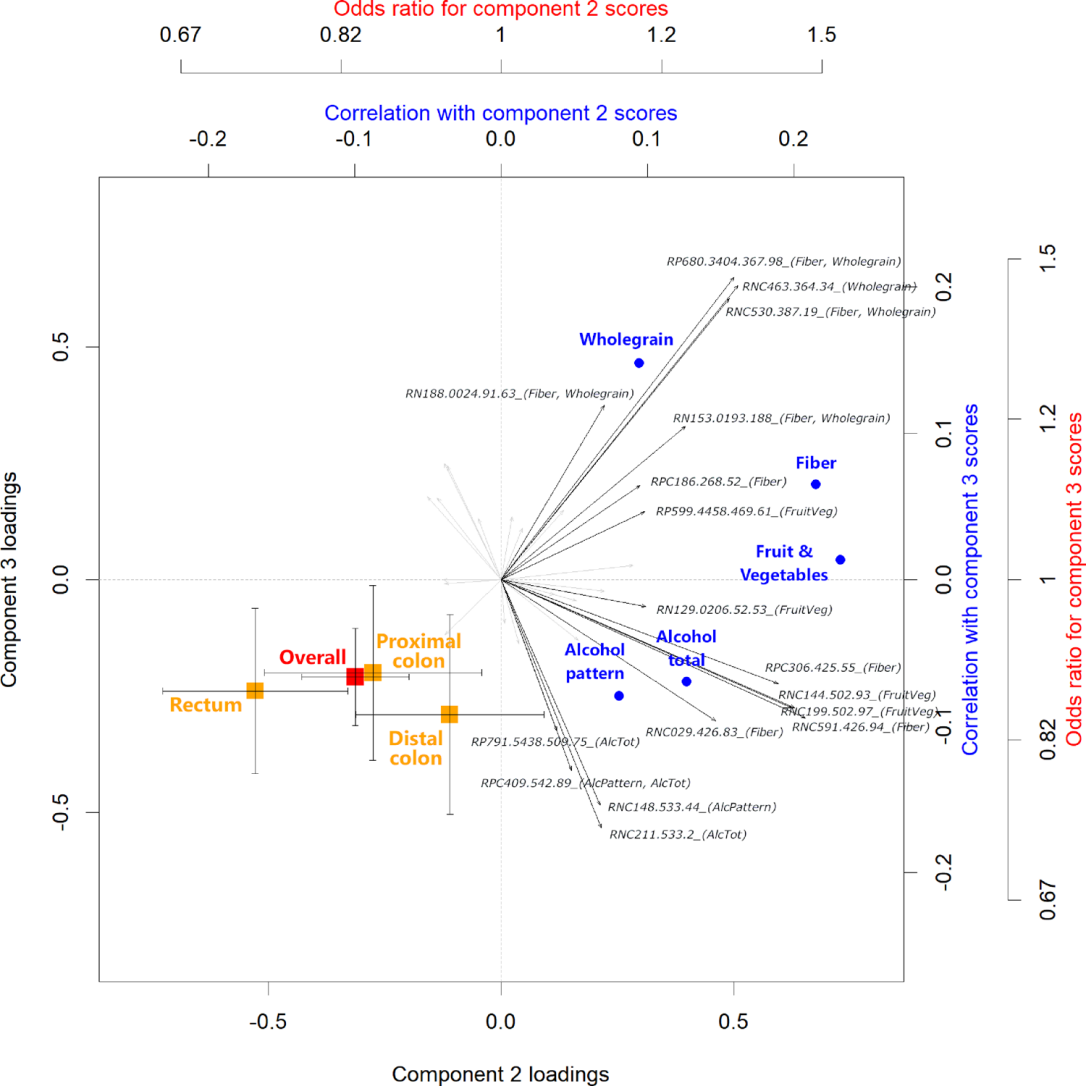
TriPlot displaying metabolite loadings (black arrows) from principal component analysis (PCA) of metabolite features selected to reflect dietary exposures (n = 36). Metabolite feature names are reported as unique identifier (characteristics reported in Suppl Table 3; most metabolite identities are unknown) followed by the dietary pattern they reflect (in parentheses). Component scores were associated to colorectal cancer risk estimated by odds ratios (in red and orange, with whiskers denoting standard error) and to dietary exposures using partial Spearman correlation (in blue), adjusted for body mass index, smoking status, recreational physical activity, educational level, total energy intake, and alcohol intake (for association to alcohol pattern/total, alcohol was not included as a confounder).

From the PCA including all 36 diet-related features, the metabolite pattern in the first component correlated with alcohol consumption (r_alcoholpattern_ = 0.45; r_alcoholamount_ = 0.44) but attenuated after adjustment for confounders (r_alcoholpattern, partial_ = 0.31; r_alcoholamount, partial_ = 0.31) and did not associate with CRC risk (OR = 1.00 (0.88–1.13), p = 0.99). The second component reflected fruit and vegetables (r_Fruitvegetables, partial_ = 0.23), fiber (r_Fiber, partial_ = 0.21) and wholegrain (r_Wholegrain, partial_ = 0.09), and associated with decreased CRC risk (OR = 0.83 (0.73–0.95), p = 0.007), more pronounced in women (OR = 0.79 (0.65–0.95), p = 0.014) than men (OR = 0.85 (0.70–1.03), p = 0.105). The association reflected predominantly rectal cancer (OR = 0.74 (0.59–0.92), p = 0.008), again more pronounced in women (OR = 0.56 (0.37–0.84), p = 0.005). The third component also reflected wholegrain (r_wholegrain, partial_ = 0.15) and associated with decreased CRC risk (OR = 0.89 (0.79–1.00), p = 0.046), but with no distinct association to cancer site or sex in subgroup analyses. A triplot of the second and third components and their associations to dietary exposures and CRC risk (overall and per site), adjusted for confounders, is shown for all participants in Fig. 2 and stratified for men and women in Suppl Fig. 1.

## Discussion

In this population-based, nested case–control study, we explored data-driven (a posteriori) dietary patterns and hypothesis-driven (a priori) dietary components in relation to the untargeted plasma metabolome and future CRC risk. Overall, associations between dietary patterns and components to either the metabolome or CRC risk were modest and mostly consistent with known associations.

Among the 12 data-driven patterns identified, only the *breakfast food* pattern, characterized by fermented milk products (low fat and 3% fat Swedish “filmjölk” and yoghurt), fiber-rich breakfast cereals, and berries (fresh and frozen) associated with a lower risk of CRC (Table 3), potentially summarizing an effect of the earlier established or probable protective dietary exposures factors, namely calcium and dietary fiber^4^. Our finding of an inverse association for dairy products and calcium intake supports that interpretation. Surprisingly, total fiber intake alone was not associated with CRC risk in this study. Since cereal fibre in specific has been shown to potentially drive the association to CRC^50^, the use of total fiber in our study may have masked a true association due to misclassification. In subgroup analysis, association to the *breakfast food* was pronounced especially for distal colon cancer and particularly in women (Table 4). Another pattern possibly describing breakfast foods (i.e., the *fruit soup and rice* pattern) showed similar reduced risk for distal colon cancer in women although it did not associate to overall CRC risk.

Given the strong scientific evidence for an increased risk of CRC with larger intakes of red and processed meat^2^, it was surprising that we did not see statistically significant associations for this in our study population, nor for women or for men separately. However, subgroup analysis further showed that the *meat* and *fast-food* patterns associated with rectal cancer risk in women, in line with other findings for association between red and processed meat and cancer in the distal colon and rectum, but not in the proximal colon^51–53^. Risk estimates for the hypothesis-driven dietary components dairy products and calcium intake also demonstrated more of the expected associations in women^51^, with largely null results in men. Although alcohol consumption has been convincingly associated with increased CRC risk^2^, we observed no such association for either men or women. The discrepancy in associations for men and women in this study could be due to differences in eating behaviour^54^, self-reporting of dietary data^55^, or have biological meaning^56^. Although we cannot distinguish which of these is the main explanatory factor for the observed differences, or if they are chance findings, sex stratification is important to conduct to gain more knowledge of sex and gender disparities in CRC risk^56^.

Stratification by anatomical tumor location also revealed some possible site-specific risk relationships. There are several known differences between proximal and distal tumors regarding epidemiology, clinical manifestation, pathology, and prognosis^57^. Patients with proximal tumors are more often older, women, have more co-morbidities, different molecular tumor characteristics, and poorer prognosis than patients with distal tumors^57^. In contrast, distal tumors more often show chromosomal instability^53^. With regards to food digestion, the proximal and distal parts of the colon have different exposures to bowel content and hence also different microbiota^58^. For site-specific dietary associations, results to date are largely inconclusive, including results for dietary fibre and wholegrain^9,51^, dairy products^51,59^ and dietary patterns^60^, although a western diet has been suggested to be associated especially with increased risk of distal colon cancer and rectal cancer^61^. Similarly, anatomical tumor site does seem to potentially modify associations between meat and CRC risk. A large study including pooled data on > 400,000 participants found a significant right-to-left trend for intake of unprocessed red meat, with risk estimates lowest for proximal colon cancer and highest for rectal cancer^53^. Subgrouping by tumor location should be a general priority in future studies of diet and CRC risk.

Among the data-driven dietary patterns, only *alcohol* associated with the untargeted plasma metabolome, while four of the hypothesis-driven dietary components (wholegrains, total fiber, fruits and vegetables, and total alcohol) associated with metabolite profiles (Table 3). These results highlight that although data-driven dietary patterns can describe eating habits in the study population, such patterns may be constituted of food items with vastly different underlying molecular profiles, not easily described using molecular techniques. Conversely, the hypothesis-driven dietary components seem to better capture specific exposures quantitatively and may be more homogenous than the data-driven patterns in terms of chemical constitutes. The associations observed between food components and metabolite profiles in our study were all foods with previously reported food-metabolites in the literature (fruit and vegetables, fibre, wholegrain, and alcohol)^62^. Similar to the *breakfast food* pattern in our study, a healthy dietary pattern characterized by higher intakes of breakfast cereal and porridge, low fat and skimmed milks, as well as potatoes, fruit and fish, was identified from a previous cluster analysis of semi-weighed food diaries^63^. However, unlike the present study, the healthy dietary pattern in that report also correlated with metabolomics profiles, based on urine samples. Although only the *breakfast food* pattern associated significantly to CRC risk in this study, the included components and direction of risk estimates for the other 11 patterns were generally in line with present dietary guidelines; for example, to eat more fruit and fiber and to limit intake of meat, saturated fat, fast food, and alcohol, while non-significant risk estimates above 1.0 for *vegetables* and *fish* patterns were somewhat conflicting against dietary guidelines^64^.

Metabolite features reflecting intakes of wholegrain and dietary fiber were inversely associated with CRC risk, which contrasts the unexpected null result for dietary intake of wholegrain and fiber^4^. In addition, both the *alcohol* pattern and total calculated intake of alcohol were associated with metabolite profiles but not with CRC risk, which was surprising^2,3^. Interestingly, a single alcohol-related metabolite (2 isotopes and an isomer) was associated with CRC risk, whereas most alcohol-related metabolites were not. While this could be a false discovery, it could also indicate subgroup dependencies in the effects of alcohol on CRC risk. Unfortunately, the metabolite of interest could not be identified, and biological interpretation is therefore not possible. In a previous study with large overlap of the same population in this study, we reported associations between metabolites and incident CRC, both novel associations and replication of previous observations^38^. Though insufficient for potential clinical implementation, such as risk stratification or precision screening, the results, together with the present findings, add to the body of evidence supporting the value of the circulating metabolome for understanding CRC risk factors and etiology^65^.

When aggregating diet-associated metabolite features in a PCA, the metabolite pattern in the first component reflected alcohol intake, captured both in the *alcohol* pattern and as total calculated intake. Similar to the CRC association calculated directly from the alcohol intake, the association of the alcohol-related metabolite profile with CRC risk was also null. The second and third components reflected metabolite patterns related to intakes of dietary fiber, fruit and vegetables, as well as wholegrain. These components indicated an association with lower CRC risk consistent with established reduced CRC risk for wholegrain and dietary fiber^2,9^. Also in this subgroup analysis, the association was more pronounced in women, but for rectal cancer, rather than distal colon cancer (Fig. 2, Suppl Fig. 1B). Interestingly, the metabolite profile reflecting dietary fiber, fruit and vegetables, and wholegrain provided CRC associations in stronger accordance with literature compared to estimates derived from self-reported dietary intake. Hence, biomarkers may have the potential to reflect dietary intake better than self-reporting, but it could also indicate that biomarkers are sensitive to many physiological processes and thus indicative of CRC risk beyond their sole reflection of specific dietary intakes. Nevertheless, self-reported and objective measures of dietary intake might have unrelated sources of bias and thus be combined to strengthen the interpretation of observational studies.

Our study had several limitations. Using self-reported dietary exposures from FFQs can introduce both random and systematic measurement errors, in particular for self-reported alcohol intake^66^. However, several validation studies, including use of biomarkers, suggested validity similar to FFQs used in other large-scale studies^34,35^. We adjusted for energy intake using the density method^67^. Several other methods exist^68^ although none are likely to sufficiently account for all measurement bias. The exclusion of participants due to insufficient self-reported data was a limitation but based on minor differences in baseline characteristics between included and excluded participants (Suppl Table 2), we consider the risk of substantial selection bias to be low. Furthermore, selection bias in the cohort has been reported to be minor^69,70^, which also supports generalizability. Sampling weights, a method sometimes used to potentially enhance representativeness in population-based studies, could be argued for but is not without its limitation and needs to be properly incorporated not to increase bias^71^. Including in the models, as we did, the variables that might account for disproportionate representation in the sample design as independent variables should at least mitigate bias in this investigation.

Another limitation was the possibility of residual confounding by covariates that we could not adjust for, such as family history of CRC and nonsteroidal anti-inflammatory drug use. Although the sample size was relatively large for the combination of pre-diagnostic dietary data and untargeted plasma metabolomics data, the statistical power for subgroup analyses was limited. In addition, most metabolite features remained unidentified, due to a combination of low intensity signals not capable of generating MS2 level data for annotation, as well as an absence of hits in reference data bases, likely reflecting that the food metabolome and exposome are still understudied. As our study was exploratory in nature, we did not account for multiple testing. Our intent in including the metabolomics analyses was to explore the circulating metabolome as a potential source of metabolic markers or marker patterns reflective of diet, not to identify possible carcinogenic metabolites stemming from the diet. Thus, we did not conduct formal mediation analyses. Such an approach might be considered in future studies but was not warranted as a post hoc analysis our investigation given the modest results.

The main strengths of this study were the population-based design, prospectively collected samples and dietary data for a recall period of one year for the participants, and high-quality sample collection and handling procedures enabling metabolomics analysis. The long follow-up from the data collection including the plasma samples to the CRC diagnosis of cases (median 11.3 years) was also advantageous, given the long carcinogenic process in CRC development. We consider the exploratory approach to be a strength of the investigation. The difficulty in establishing dietary risk factors for CRC, despite decades of epidemiological research, makes it clear that novel approaches are useful and may have promise for improving the depth of understanding of the link between diet and CRC, or as in this study confirming some of the earlier known dietary risk factors.

Despite strong agreement both on the importance of diet in CRC development and on the value of dietary pattern analysis in assessing the relation between diet and disease, the evidence to date is still insufficient to form convincing conclusions and guidelines for CRC risk prevention^4^. In this context, our study demonstrates the potential of incorporating both innovative data-driven techniques, comparisons of methods producing dietary patterns, as well as biomarker identification of potential diet-disease features, toward improving the understanding of CRC etiology. The use of data-driven dietary pattern analyses has been suggested as a complement to more traditional, hypothesis-driven pattern analyses, to help capture the complexity of diet^13,72^. In the present study, we used a robust validation approach combining exploratory and confirmatory factor analysis in a repeated random half split procedure, which identified 12 data-driven food patterns, that were considered relevant. Previous investigations have tended to present fewer latent variables^19,73^, arguably corresponding to overarching dietary patterns. Here, the higher-resolution factorization had both a better reproducibility of the diet variable composition and a better fit to the data than factorization with a lower number of latent variables.

In conclusion, in this population-based nested case–control study, we identified 12 robust data-driven dietary patterns, of which the *breakfast food* pattern associated with overall CRC risk. Observed inverse associations for the a priori known components dietary calcium and dairy foods strengthened the results from the exploratory analysis producing dietary patterns. Some possible site-specific relations were found; the *breakfast food* pattern was associated with reduced risk of distal colon cancer, particularly in women, as was the *fruit soup and rice* pattern, and the *meat* and *fast-food* patterns were associated with rectal cancer in women. Associations with metabolite profiles were observed for a priori components wholegrain, fiber, alcohol, and fruits and vegetables, and for data driven patterns only for the *alcohol* pattern. In accordance with earlier reported diet-CRC association, three metabolites, reflecting fiber and wholegrain, and fruit and vegetable intake, showed nominally significant associations to decreased CRC risk, whereas one alcohol-related metabolite showed a nominally signification association to increased CRC risk. When aggregated, the diet-related metabolite profiles indicated inverse CRC associations for dietary fiber, fruit and vegetables, and wholegrain, especially for female rectal cancer.

### Supplementary Information

Supplementary Information.

## Data Availability

The data generated in this study are not publicly available due to Swedish Authority for Privacy Protection regulations (the national supervisory authority under the European General Data Protection Regulation, GDPR). Data may be available upon reasonable request to the corresponding author.
