# Supplementary material for: Dietary patterns, untargeted metabolite profiles and their association with colorectal cancer risk
Source: Sci Rep. 2024 Jan 26;14:2244. doi: 10.1038/s41598-023-50567-6 (PMC10817924; doi:10.1038/s41598-023-50567-6)
Supplement: Supplementary file 1 — Supplementary Information. [file 41598_2023_50567_MOESM1_ESM.docx]

# **SUPPLEMENTARY MATERIAL**

Supplementary material containing information about parameters used for the R packages used for analyses of metabolomics data (Suppl Table 1), baseline characteristics of included and excluded participants for comparison (Suppl Table 2), dietary variable loadings from factor analysis (Suppl Table 3, odds ratios (ORs) for data-driven dietary patterns showing no significant sex- or tumor-site-specific associations with colorectal cancer risk (Suppl Table 4), information about metabolite features (Suppl Table 5), and TriPlots for a) men and b) women displaying metabolite loadings from principal component analyses (PCAs) of metabolites features selected to reflect dietary exposures (Suppl Fig 1).

| **Supplementary Table 1.** Parameters used for the R packages XSMS. RAMClustR and the software Sirius+CSI:FingerID. |
| --- |
| **Parameters used for XCMS^a^** |
| - Reverse phase Negative (RN): CentWaveParam(peakwidth = c(7. 60). noise=500. ppm=22. mzdiff=0.0014. prefilter=c(3. 5000). integrate=1); PeakGroupsParam(minfrac=0.95. smooth="loess". span=0.4. family="gaussian"); PeakDensityParam(minfrac=0.4. bw=1.5. binSize=0.015). |
| - Reverse phase Positive (RP): CentWaveParam(peakwidth=c(7.5. 62). noise=500. ppm=20. mzdiff=-0.003. prefilter=c(3. 5000). integrate=1); PeakGroupsParam(minfrac=0.95. smooth="loess". span=0.4. family="gaussian"); PeakDensityParam(minfrac=0.4. bw=1.5. binSize=0.015). |
| **Parameters used for RAMClustR** |
| - The parameters were manually optimized as follows: st=0.5. sr=0.35. which resulted in 775 and 820 clusters for RN and RP modes. respectively. For each cluster. feature with the highest intensity that was considered to be the representative of this cluster and together with 1616 and 1824 singletons (i.e. features not included in any cluster) for the two modes were subsequently used in multivariate modelling.   **Parameters used for Sirius+CSI:FingerID v4.8.2**   - SIRIUS + ZODIAC + CSI:FingerID used for all predictions. All set to default settings except for “Consider only formulas in DBs:” option in the Sirius settings which was set to “Bio Database”.   **Parameters used for MetFrag**   - Database settings: Pubchem Lite. Search ppm = 5 - Candidate Filter & Score Settings: Exact spectral similarity (MoNA) - Fragmentation Settings & Processing: Default settings   **Parameters used for HMDB**   - DPPM = 10. Adducts = M+H. M-H   **Parameters used for in-house library**   - Settings: DPPM = 5. dRT = 15s. mzWeight = 0. intWeight = 1 - Cut-offs: Cosine sim. score = 0.9. n matching peaks: 2 |
| ^a^ Other parameters in XCMS were default values. |

| **Supplementary Table 2. Baseline characteristics of excluded 330 cases and 330 controls due to insufficient data and the included 680 cases and 680 controls.** | | | | | | | | |
| --- | --- | --- | --- | --- | --- | --- | --- | --- |
|  | **EXKLUDED FROM STUDY** | | | | **INCLUDED IN STUDY** | | | |
| **Variable** | **Total**  **n=660** | **Cases**  **n=330** | **Controls**  **n=330** | **Missing.**  **n (%)** | **Total**  **n=1360** | **Cases**  **n= 680** | **Controls.**  **n= 680** | **Missing.**  **n (%)** |
| **Age at baseline. years. median (IQR)** | 50.4 (49.9-60.0) | 50.5 (49.9-60.0) | 50.3 (49.9-60.0) | - | 59.7 (50.0-60.0) | 59.7 (49.9-60.0) | 59.7 (50.0-60.0) | - |
| **Age at baseline. years. mean (sd)** | 53.2 (8.3) | 53.2 (8.3) | 53.2 (8.3) |  | 54.6 (7.7) | 54.6 (7.8) | 54.6 (7.7) | - |
| **Follow-up time. years. median (IQR)** | 12.7 (9.2-17.7) | 12.6 (9.2-17.7) | 12.8 (9.17.7) | - | 11.3 (6.4-15.6) | 11.3 (6.4-15.5) | 11.3 (6.5-15.7) | - |
| **Sex. n (%)** |  |  |  | - |  |  |  | - |
| Men | 362 (54.8) | 181 (54.8) | 181 (54.8) |  | 688 (50.6) | 344 (50.6) | 344 (50.6) |  |
| Women | 298 (45.2) | 149 (45.2) | 149 (45.2) |  | 672 (49.4) | 336 (49.4) | 336 (49.4) |  |
| **Cohort. n (%)** |  |  |  |  |  |  |  | - |
| VIP | 614 (93.0) | 307 (93.0) | 307 (93.0) | - | 1242 (91.3) | 621 (91.3) | 621 (91.3) |  |
| MONICA | 46 (7.0) | 23 (7.0) | 23 (7.0) |  | 118 (8.7) | 59 (8.7) | 59 (8.7) |  |
| **BMI kg/m^2^. n (%)** |  |  |  | 9 (1.4) |  |  |  | 8 (0.4) |
| <25 normal weight | 286 (43.3) | 133 (40.3) | 153 (46.4) |  | 527 (38.8) | 248 (36.5) | 279 (41.0) |  |
| 25-30 overweight | 279 (42.3) | 148 (44.8) | 131 (39.7) |  | 606 (44.6) | 311 (45.7) | 295 (43.4) |  |
| >30 obese | 86 (13.0) | 45 (13.6) | 41 (12.4) |  | 219 (16.1) | 117 (17.2) | 102 (15.0) |  |
| Missing | 9 (1.4) | 4 (1.2) | 5 (1.5) |  | 8 (0.4) | 4 (0.4) | 4 (0.74) |  |
| **BMI kg/m^2^. mean (sd)** | 25.9 (4.0) | 26.1 (3.4) | 25.8 (4.4) |  | 26.4 (4.0) | 26.6 (4.1) | 26.2 (3.8) |  |
| **Smoking status. n (%)** |  |  |  | 27 (4.1) |  |  |  | 14 (1.0) |
| Never smoker | 266 (40.4) | 121 (36.7) | 145 (44.1) |  | 572 (42.1) | 272 (40.0) | 300 (44.1) |  |
| Ex-smoker | 207 (31.4) | 114 (34.5) | 93 (28.3) |  | 476 (35.0) | 248 (36.5) | 228 (33.5) |  |
| Current smoker | 159 (24.1) | 85 (25.8) | 74 (22.5) |  | 298 (21.9) | 151 (22.2) | 147 (21.6) |  |
| Missing | 27 (4.1) | 10 (3.0) | 17 (5.2) |  | 14 (1.0) | 9 (1.3) | 5 (0.7) |  |
| **Recreational physical activity level. n (%)** |  |  |  | 111 (16.8) |  |  |  | 17 (1.3) |
| None | 239 (36.2) | 130 (39.4) | 109 (33.0) |  | 575 (42.4) | 298 (43.9) | 277 (40.9) |  |
| Low (occasionally) | 135 (20.5) | 56 (17.0) | 79 (23.9) |  | 347 (25.6) | 172 (25.3) | 175 (25.8) |  |
| Medium (1-3 times/w) | 148 (22.4) | 78 (23.6) | 70 (21.2) |  | 356 (26.2) | 175 (25.8) | 181 (26.7) |  |
| High (>3 times/w with higher intensity) | 27 (4.1) | 14 (4.2) | 13 (3.9) |  | 65 (4.8) | 29 (4.3) | 36 (5.3) |  |
| Missing | 111 (16.8) | 52 (15.8) | 59 (17.9) |  | 17 (1.3) | 6 (0.9) | 11 (1.6) |  |
| **Educational level. n (%)** |  |  |  | 27 (4.1) |  |  |  | 9 (0.7) |
| Elementary school | 281 (42.6) | 139 (42.1) | 142 (43.0) |  | 511 (37.6) | 245 (36.0) | 266 (39.1) |  |
| Secondary school | 255 (38.6) | 126 (38.2) | 129 (39.1) |  | 600 (44.1) | 315 (46.3) | 285 (41.9) |  |
| Post-secondary school | 97 (14.7) | 50 (15.2) | 47 (14.2) |  | 240 (17.6) | 115 (16.9) | 125 (18.4) |  |
| Missing | 27 (4.1) | 15 (4.5) | 12 (3.6) |  | 9 (0.7) | 5 (0.7) | 4 (0.6) |  |
| **Civil status. n (%)** |  |  |  | 11 (1.7) |  |  |  | 17 (1.3) |
| Unmarried | 64 (9.7) | 24 (7.3) | 40 (12.1) |  | 101 (7.4) | 44 (6.5) | 57 (8.4) |  |
| Married or cohabitant | 533 (80.8) | 276 (83.6) | 257 (77.9) |  | 1091 (80.2) | 546 (80.3) | 545 (80.1) |  |
| Separated | 26 (3.9) | 9 (2.7) | 17 (5.2) |  | 98 (7.2) | 46 (6.8) | 52 (7.6) |  |
| Widow/widower | 26 (3.9) | 12 (3.6) | 14 (4.2) |  | 53 (3.9) | 37 (5.4) | 16 (2.4) |  |
| Missing | 11 (1.7) | 9 (2.7) | 2 (0.6) |  | 17 (1.3) | 7 (1.0) | 10 (1.5) |  |
| **Alcohol intake. g/day. n (%)** |  |  |  | 213 (32.3) |  |  |  | - |
| Zero intake | 31 (6.9) | 18 (8.0) | 13 (5.9) |  | 121 (8.9) | 57 (8.4) | 64 (9.4) |  |
| Below median (sex-specific) | 209 (46.8) | 102 (45.1) | 107 (48.4) |  | 559 (41.1) | 295 (43.4) | 264 (38.8) |  |
| Above median (sex-specific) | 207 (46.3) | 106 (45.1) | 101 (45.7) |  | 680 (50.0) | 328 (48.2) | 352 (51.8) |  |
| **Alcohol intake. g/day. mean (sd)** | 4.0 (5.1) | 4.2 (5.4) | 3.8 (4.7) |  | 4.0 (4.8) | 4.1 (5.1) | 3.9 (4.4) |  |
| **Energy intake. kcal/day. mean (sd)** | 1770 (878) | 1778 (926) | 1762 (825) | 294 (44.5) | 1704 (637) | 1683 (644) | 1724 (29) | - |
| IQR. interquartile range; BMI. body mass index. VIP. Västerbotten Intervention Programme; MONICA. Multinational Monitoring of Trends and Determinants in Cardiovascular Disease | | | |  |  |  |  |  |

| **Supplementary Table 3. Dietary variable loadings showing the direction of intake in relation to the factor scores.** | | | | | | | | | | | | |
| --- | --- | --- | --- | --- | --- | --- | --- | --- | --- | --- | --- | --- |
|  | *meat* | *alcohol* | *snack* | *fish* | *breakfast* | *fastfood* | *fruitrice* | *spread* | *fullfat* | *smoked* | *veg* | *breadspread* |
| **Factor Scores** |  |  |  |  |  |  |  |  |  |  |  |  |
| mean | -9.88E-18 | 7.26E-17 | -1.06E-17 | 7.19E-17 | 3.43E-17 | 4.27E-17 | 1.96E-17 | -8.67E-17 | 2.32E-18 | 1.90E-17 | -1.04E-17 | -3.93E-17 |
| SD | 0.85608 | 0.807059 | 0.765691 | 0.806943 | 1.036125 | 0.801744 | 0.871962 | 1.009214 | 0.825219 | 0.718892 | 0.802563 | 0.69075 |
|  |  |  |  |  |  |  |  |  |  |  |  |  |
| **Factor Loadings** |  |  |  |  |  |  |  |  |  |  |  |  |
| Ground meat dishes | 0.582253 | 0 | 0 | 0 | 0 | 0 | 0 | 0 | 0 | 0 | 0 | 0 |
| Beef stew | 0.744142 | 0 | 0 | 0 | 0 | 0 | 0 | 0 | 0 | 0 | 0 | 0 |
| Steak, pork chop | 0.617724 | 0 | 0 | 0 | 0 | 0 | 0 | 0 | 0 | 0 | 0 | 0 |
| Beer, 2.8 & 3.5% alcohol | 0 | 0.374716 | 0 | 0 | 0 | 0 | 0 | 0 | 0 | 0 | 0 | 0 |
| Beer, 4.5% alcohol | 0 | 0.61172 | 0 | 0 | 0 | 0 | 0 | 0 | 0 | 0 | 0 | 0 |
| Wine | 0 | 0.440932 | 0 | 0 | 0 | 0 | 0 | 0 | 0 | 0 | 0 | 0 |
| Spirits, 40% alcohol | 0 | 0.655938 | 0 | 0 | 0 | 0 | 0 | 0 | 0 | 0 | 0 | 0 |
| Wheat buns, rusk | 0 | 0 | 0.406862 | 0 | 0 | 0 | 0 | 0 | 0 | 0 | 0 | 0.249308 |
| Sweets. e.g., chocolate. candy | 0 | 0 | 0.432125 | 0 | 0 | 0 | 0 | 0 | 0 | 0 | 0 | 0 |
| Cakes, cookies., pastry | 0 | 0 | 0.663487 | 0 | 0 | 0 | 0 | 0 | 0 | 0 | 0 | 0 |
| Chips, popcorn, salted nuts, etc. | 0 | 0 | 0.232829 | 0 | 0 | 0 | 0 | 0 | 0 | 0 | 0 | 0 |
| Lean fish (perch, cod) | 0 | 0 | 0 | 0.363001 | 0 | 0 | 0 | 0 | 0 | 0 | 0 | 0 |
| Fatty and lean fish (herring, white fish, salmon) | 0 | 0 | 0 | 0.727164 | 0 | 0 | 0 | 0 | 0 | 0 | 0 | 0 |
| Salted fish (herring) | 0 | 0 | 0 | 0.491252 | 0 | 0 | 0 | 0 | 0 | 0 | 0 | 0 |
| Soured milk, yoghurt. 3% fat | 0 | 0 | 0 | 0 | 0.322769 | 0 | 0 | 0 | 0 | 0 | 0 | 0 |
| Soured milk, yoghurt. 0.5% fat | 0 | 0 | 0 | 0 | 0.338657 | 0 | 0 | 0 | 0 | 0 | 0 | 0 |
| Fibre-rich cereals. e.g. musli | 0 | 0 | 0 | 0 | 1.03513 | 0 | 0 | 0 | 0 | 0 | 0 | 0 |
| Berries, fresh or deep frozen | 0 | 0 | 0 | 0 | 0.181237 | 0 | 0 | 0 | 0 | 0 | 0 | 0 |
| Pizza | 0 | 0 | 0 | 0 | 0 | 0.392798 | 0 | 0 | 0 | 0 | 0 | 0 |
| Bacon. pork belly, ham | 0 | 0 | 0 | 0 | 0 | 0.542321 | 0 | 0 | 0 | 0 | 0 | 0 |
| Sausage as a main course | 0 | 0 | 0 | 0 | 0 | 0.523828 | 0 | 0 | 0 | 0 | 0 | 0 |
| Hamburger | 0 | 0 | 0 | 0 | 0 | 0.515582 | 0 | 0 | 0 | 0 | 0 | 0 |
| Rose hip soup, fruit syrup soup, thickened fruit syrup and water | 0 | 0 | 0 | 0 | 0 | 0 | 0.382996 | 0 | 0 | 0 | 0 | 0 |
| Rice | 0 | 0 | 0 | 0 | 0 | 0 | 0.857216 | 0 | 0 | 0 | 0 | 0 |
| Butter, 80% fat, on sandwich | 0 | 0 | 0 | 0 | 0 | 0 | 0 | 1.00892 | 0 | 0 | 0 | 0 |
| Margarine, 40-60% fat on sandwich | 0 | 0 | 0 | 0 | 0 | 0 | 0 | -0.54578 | 0 | 0 | 0 | 0.315746 |
| Butter on sandwich | 0 | 0 | 0 | 0 | 0 | 0 | 0 | 0 | 0.332668 | 0 | 0 | 0 |
| Butter in cooking | 0 | 0 | 0 | 0 | 0 | 0 | 0 | 0 | 0.807319 | 0 | 0 | 0 |
| Milk, 3% fat | 0 | 0 | 0 | 0 | 0 | 0 | 0 | 0 | 0.167744 | 0 | 0 | 0 |
| Meat as sandwich topping | 0 | 0 | 0 | 0 | 0 | 0 | 0 | 0 | 0 | 0.54503 | 0 | 0 |
| Smoked fish/meat | 0 | 0 | 0 | 0 | 0 | 0 | 0 | 0 | 0 | 0.394715 | 0 | 0 |
| Root vegetables, carrots | 0 | 0 | 0 | 0 | 0 | 0 | 0 | 0 | 0 | 0 | 0.671849 | 0 |
| Tomato, cucumber | 0 | 0 | 0 | 0 | 0 | 0 | 0 | 0 | 0 | 0 | 0.640031 | 0 |
| Dark crisp bread, e.g., rye | 0 | 0 | 0 | 0 | 0 | 0 | 0 | 0 | 0 | 0 | 0 | 0.56576 |
| Hard cheese, 10-17 % fat | 0 | 0 | 0 | 0 | 0 | 0 | 0 | 0 | 0 | 0 | 0 | 0.265295 |
|  |  |  |  |  |  |  |  |  |  |  |  |  |
| SD. standarddeviation | | | | | | | | | | | | |

| **Supplementary Table 4. Odds ratios (ORs) for data-driven dietary patterns showing no significant sex- or tumor-site-specific associations with colorectal cancer risk.** | | | | | | | | | |
| --- | --- | --- | --- | --- | --- | --- | --- | --- | --- |
|  | **All** | | | **Women** | | | **Men** | | |
| **Dietary factors ^a.b^** | **n** | **OR (95% CI)** | **P** | **n** | **OR (95% CI)** | **P** | **n** | **OR (95% CI)** | **P** |
|  |  |  |  |  |  |  |  |  |  |
| **Bread with spreads** |  |  |  |  |  |  |  |  |  |
| Proximal colon | 454 | 0.96 (0.75-1.21) | 0.72 | 272 | 0.82 (0.58-1.16) | 0..27 | 182 | 1.17 (0.79-1.73) | 0.43 |
| Distal colon | 460 | 0.96 (0.77-1.19) | 0.69 | 210 | 1.20 (0.84-1.70) | 0.32 | 250 | 0.82 (0.59-1.13) | 0.23 |
| Rectum | 612 | 0.97 (0.79-1.19) | 0.77 | 270 | 0.78 (0.55-1.09) | 0.14 | 342 | 1.09 (0.83-1.43) | 0.53 |
|  |  |  |  |  |  |  |  |  |  |
| **Smoked** |  |  |  |  |  |  |  |  |  |
| Proximal colon | 454 | 0.94 (0.72-1.22) | 0.63 | 272 | 0.78 (0.55-1.10) | 0.15 | 182 | 1.40 (0.83-2.36) | 0.21 |
| Distal colon | 460 | 0.96 (0.77-1.21) | 0.75 | 210 | 1.10 (0.80-1.53) | 0.55 | 250 | 0.79 (0.55-1.14) | 0.21 |
| Rectum | 612 | 0.93 (0.75-1.15) | 0.49 | 270 | 0.96 (0.68-1.36) | 0.82 | 342 | 0.93 (0.70-1.23) | 0.61 |
|  |  |  |  |  |  |  |  |  |  |
| **Snacks and sweets** |  |  |  |  |  |  |  |  |  |
| Proximal colon | 454 | 1.13 (0.90-1.42) | 0.30 | 272 | 1.30 (0.94-1.80) | 0.12 | 182 | 1.04 (0.68-1.58) | 0.86 |
| Distal colon | 460 | 0.90 (0.71-1.14) | 0.37 | 210 | 1.05 (0.74-1.49) | 0.79 | 250 | 0.76 (0.53-1.10) | 0.14 |
| Rectum | 612 | 0.97 (0.79-1.19) | 0.76 | 270 | 1.03 (0.73-1.45) | 0.87 | 342 | 0.93 (0.71-1.23) | 0.63 |
|  |  |  |  |  |  |  |  |  |  |
| **Spreads** |  |  |  |  |  |  |  |  |  |
| Proximal colon | 454 | 1.02 (0.82-1.28) | 0.84 | 272 | 1.05 (0.77-1.43) | 0.75 | 182 | 0.95 (0.65-1.40) | 0.81 |
| Distal colon | 460 | 0.95 (0.78-1.16) | 0.63 | 210 | 1.06 (0.79-1.43) | 0.70 | 250 | 0.85 (0.63-1.14) | 0.27 |
| Rectum | 612 | 1.05 (0.88-1.25) | 0.59 | 270 | 1.10 (0.83-1.46) | 0.51 | 342 | 1.01 (0.79-1.29) | 0.95 |
|  |  |  |  |  |  |  |  |  |  |
| **Vegetables** |  |  |  |  |  |  |  |  |  |
| Proximal colon | 454 | 1.06 (0.85-1.32) | 0.58 | 272 | 1.09 (0.83-1.45) | 0.53 | 182 | 1.07 (0.71-1.62) | 0.75 |
| Distal colon | 460 | 1.00 (0.81-1.25) | 0.98 | 210 | 0.91 (0.65-1.28) | 0.59 | 250 | 1.14 (0.82-1.57) | 0.45 |
| Rectum | 612 | 0.97 (0.80-1.18) | 0.77 | 270 | 1.06 (0.78-1.44) | 0.70 | 342 | 0.90 (0.68-1.18) | 0.45 |
|  |  |  |  |  |  |  |  |  |  |
| **Full fat products** |  |  |  |  |  |  |  |  |  |
| Proximal colon | 454 | 1.05 (0.85-1.30) | 0.63 | 272 | 1.18 (0.87-1.59) | 0.28 | 182 | 0.88 (0.60-1.27) | 0.49 |
| Distal colon | 460 | 1.15 (0.94-1.42) | 0.18 | 210 | 1.24 (0.89-1.71) | 0.20 | 250 | 1.12 (0.83-1.52) | 0.46 |
| Rectum | 612 | 0.98 (0.81-1.17) | 0.79 | 270 | 1.01 (0.75-1.35) | 0.95 | 342 | 0.92 (0.72-1.18) | 0.51 |
|  |  |  |  |  |  |  |  |  |  |
| **Fish** |  |  |  |  |  |  |  |  |  |
| Proximal colon | 454 | 1.08 (0.86-1.36) | 0.50 | 272 | 0.92 (0.68-1.25) | 0.60 | 182 | 1.37 (0.92-2.05) | 0.12 |
| Distal colon | 460 | 1.09 (0.88-1.35) | 0.43 | 210 | 0.99 (0.72-1.36) | 0.95 | 250 | 1.20 (0.87-1.68) | 0.27 |
| Rectum | 612 | 1.09 (0.91-1.30) | 0.37 | 270 | 1.11 (0.81-1.51) | 0.52 | 342 | 1.08 (0.84-1.37) | 0.56 |
|  |  |  |  |  |  |  |  |  |  |
| **Alcohol ^c^** |  |  |  |  |  |  |  |  |  |
| Proximal colon | 454 | 1.07 (0.88-1.31) | 0.48 | 272 | 1.00 (0.75-1.32) | 0.99 | 182 | 1.22 (0.88-1.68) | 0.24 |
| Distal colon | 460 | 1.12 (0.91-1.38) | 0.29 | 210 | 1.16 (0.86-1.57) | 0.33 | 250 | 1.10 (0.80-1.50) | 0.55 |
| Rectum | 612 | 1.12 (0.93-1.34) | 0.24 | 270 | 1.11 (0.81-1.51) | 0.52 | 342 | 1.16 (0.91-1.48) | 0.22 |
|  |  |  |  |  |  |  |  |  |  |
| ^a^ Energy adjusted using the energy-density method  ^b^ Adjusted for potential confounders; BMI kg/m^2^. smoking (never-/ex-/current smoker). physical activity (no/low/medium/high). education (elementary school/secondary school /post-secondary school). total energy intake. kcal/day. and alcohol (non-consumers/below sex-specific median/above sex-specific median intake).  ^c^ Adjusted for the same potential confounders as for all other dietary exposures. except alcohol. | | | | | | | | | |

**Supplementary Table 5**. Metabolite features associated with diet and their association to colorectal cancer risk. adjusted for body mass index. smoking status. recreational physical activity. educational level. total energy intake. and alcohol intake (for association to alcohol pattern/total. alcohol was not included as a confounder). Annotation performed according to the Schymanski scale [1]. Metabolite features were selected from random forest analysis and further adjusted for confounders in partial Spearman correlation analysis (limited to r>0.1. fdr<0.05). Features with same group letter were manually assessed to reflect the same metabolite (cross-sample correlation (r>0.7). retention time similarity and peak shape).

|  |  |  |  |  |  |  | **Partial Spearman correlation with diet** | | | | | **CRC association** |  |
| --- | --- | --- | --- | --- | --- | --- | --- | --- | --- | --- | --- | --- | --- |
| **Feature name** | **ID LVL** | **Annotation** | **Group** | **mode** | **RT (s)** | **m/z main (fragments)** | **AlcFact** | **AlcSum** | **Fiber** | **WG** | **FruitVeg** | **OR** | **p** |
| *Alcohol* |  |  |  |  |  |  |  |  |  |  |  |  |  |
| RP176.128.45.79_(AlcTot) | 4 | [C8H17NO3+H]+ |  | RP | 45.79 | 176.1280 |  | 0.20 |  |  |  | 1.06 (0.93-1.22) | 0.36 |
| RP231.0838.56.95_(AlcPattern. AlcTot) | 4 | [C8H16O6+Na]+ |  | RP | 56.95 | 231.0838 | 0.22 | 0.26 |  |  |  | 1.01 (0.90-1.13) | 0.82 |
| RPC253.152.8_(AlcPattern. AlcTot) | 5 | Unknown |  | RP | 152.80 | 247.1441 (60.0806. 248.1474) | 0.18 | 0.17 |  |  |  | 0.98 (0.87-1.11) | 0.74 |
| RN175.0141.160.45_(AlcPattern. AlcTot) | 5 | Unknown |  | RN | 160.45 | 175.0141 | 0.19 | 0.18 |  |  |  | 1.08 (0.94-1.23) | 0.27 |
| RNC424.277.8_(AlcPattern) | 3 | Dihydromorphine glucuronide | A | RN | 277.80 | 462.1766 (463.1800) | 0.20 |  |  |  |  | 0.95 (0.84-1.07) | 0.41 |
| RPC450.279.63_(AlcPattern. AlcTot) | 3 | Dihydromorphine glucuronide | A | RP | 279.63 | 464.1916 (465.1957) | 0.20 | 0.19 |  |  |  | 0.91 (0.80-1.03) | 0.13 |
| **RN224.0623.281.9_(AlcPattern. AlcTot)** | **5** | **Unknown (isomer)** | **B^a^** | **RN** | **281.90** | **224.0623** | **0.20** | **0.20** |  |  |  | **1.14 (1.00-1.29)** | **0.04** |
| **RN224.0623.310.47_(AlcPattern)** | **5** | **Unknown (double negative charge)** | **B** | **RN** | **310.47** | **224.0623** | **0.11** |  |  |  |  | **1.17 (1.02-1.34)** | **0.03** |
| **RN224.5639.310.99_(AlcPattern. AlcTot)** | **5** | **Unknown (+1 isotope)** | **B** | **RN** | **310.99** | **224.5639** | **0.12** | **0.11** |  |  |  | **1.17 (1.02-1.34)** | **0.03** |
| RN398.9365.345_(AlcPattern) | 5 | Unknown |  | RN | 345.00 | 398.9365 | 0.11 |  |  |  |  | 1.01 (0.88-1.14) | 0.93 |
| RN499.2914.350.6_(AlcPattern. AlcTot) | 3 | Glucopyranosyloxy-hydroxylabdanenoic acid | | RN | 350.60 | 499.2914 | 0.23 | 0.23 |  |  |  | 1.01 (0.89-1.15) | 0.83 |
| RP332.2614.364.87_(AlcPattern. AlcTot) | 5 | Unknown |  | RP | 364.87 | 332.2614 | 0.20 | 0.21 |  |  |  | 0.94 (0.82-1.09) | 0.42 |
| RPC538.370.19_(AlcPattern. AlcTot) | 3 | Piperettine |  | RP | 370.19 | 312.1591 (334.1415) | 0.21 | 0.18 |  |  |  | 0.89 (0.79-1.01) | 0.08 |
| RN487.3283.390.65_(AlcPattern) | 5 | Unknown |  | RN | 390.65 | 487.3283 | 0.15 |  |  |  |  | 0.99 (0.87-1.13) | 0.87 |
| RP791.5438.509.75_(AlcTot) | 5 | Unknown |  | RP | 509.75 | 791.5438 |  | 0.11 |  |  |  | 1.02 (0.89-1.17) | 0.75 |
| RNC211.533.2_(AlcTot) | 4 | [C58H75N3O8-H]- | C | RN | 533.20 | 940.5501 (875.5712. 941.5531. 942.5555) |  | 0.19 |  |  |  | 1.01 (0.83-1.21) | 0.96 |
| RNC148.533.44_(AlcPattern) | 5 | Unknown | C | RN | 533.44 | 1008.5370 (462.1766. 463.1800. 862.5345. 930.5215. 1009.5400. 1010.5420) | 0.16 |  |  |  |  | 1.08 (0.89-1.32) | 0.45 |
| RPC409.542.89_(AlcPattern. AlcTot) | 5 | Unknown | C | RP | 542.89 | 850.8157 (851.5564) | 0.14 | 0.15 |  |  |  | 1.06 (0.88-1.27) | 0.53 |
|  |  |  |  |  |  |  |  |  |  |  |  |  |  |
| *Fiber and Wholegrain* |  |  |  |  |  |  |  |  |  |  |  |  |  |
| RP160.0969.42.53_(Fiber) | 5 | Unknown |  | RP | 42.53 | 160.0969 |  |  | 0.16 |  |  | 0.92 (0.82-1.04) | 0.18 |
| **RN188.0024.91.63_(Fiber. Wholegrain)** | **3** | **Aminophenol sulphate** |  | **RN** | **91.63** | **188.0024** |  |  | **0.19** | **0.23** |  | **0.82 (0.72-0.93)** | **0.00** |
| RN153.0193.188_(Fiber. Wholegrain) | 2 | Dihydroxybenzoic acid |  | RN | 188.00 | 153.0193 |  |  | 0.14 | 0.18 |  | 0.92 (0.82-1.04) | 0.19 |
| **RPC186.268.52_(Fiber)** | **5** | **Unknown** |  | **RP** | **268.52** | **130.0650 (131.0685. 189.0785. 190.0859)** |  |  | **0.12** |  |  | **0.88 (0.78-1.00)** | **0.04** |
| RNC463.364.34_(Wholegrain) | 3 | Pubchem: 127252567 | D | RN | 364.34 | 656.3437 (657.3470) |  |  |  | 0.16 |  | 0.93 (0.83-1.04) | 0.22 |
| RP680.3404.367.98_(Fiber. Wholegrain) | 5 | Unknown | D | RP | 367.98 | 680.3404 |  |  | 0.15 | 0.17 |  | 0.93 (0.83-1.04) | 0.19 |
| RNC530.387.19_(Fiber. Wholegrain) | 4 | [C32H49N7O8-H]- | D | RN | 387.19 | 658.3591 (659.3628) |  |  | 0.12 | 0.14 |  | 1.01 (0.90-1.13) | 0.86 |
| RPC306.425.55_(Fiber) | 5 | Unknown |  | RP | 425.55 | 329.2471 (330.2507. 374.2148) |  |  | 0.10 |  |  | 0.93 (0.81-1.06) | 0.28 |
| RNC029.426.83_(Fiber) | 3 | hydroxy-hydroxymethylhexadecanoate |  | RN | 426.83 | 379.1586 (377.1640. 380.1614. 398.1326. 400.1307. 415.2078. 416.2111. 717.4295. 718.4329) |  |  | 0.11 |  |  | 0.94 (0.81-1.08) | 0.37 |
| RNC591.426.94_(Fiber) | 4 | [C20H28O10-H]- |  | RN | 426.94 | 427.1627 (456.1810) |  |  | 0.10 |  |  | 0.98 (0.82-1.17) | 0.84 |
|  |  |  |  |  |  |  |  |  |  |  |  |  |  |
| *Fruit and Vegetables* |  |  |  |  |  |  |  |  |  |  |  |  |  |
| **RN129.0206.52.53_(FruitVeg)** | **4** | **[C5H6O4-H]-** |  | **RN** | **52.53** | **129.0206** |  |  |  |  | **0.11** | **0.85 (0.74-0.98)** | **0.02** |
| RPC369.55.93_(FruitVeg) | 5 | Unknown |  | RP | 55.93 | 130.0861 (84.0805. 131.0893) |  |  |  |  | 0.18 | 0.93 (0.82-1.06) | 0.28 |
| RN151.0401.197.94_(FruitVeg) | 5 | Unknown |  | RN | 197.94 | 151.0401 |  |  |  |  | 0.10 | 0.94 (0.84-1.05) | 0.29 |
| RN179.0714.340.69_(FruitVeg) | 3 | Methoxyphenylpropanoic acid |  | RN | 340.69 | 179.0714 |  |  |  |  | 0.11 | 0.98 (0.86-1.11) | 0.69 |
| RN469.3321.409.22_(FruitVeg) | 4 | [C30H46O4-H]- |  | RN | 409.22 | 469.3321 |  |  |  |  | 0.11 | 1.05 (0.94-1.18) | 0.37 |
| RP599.4458.469.61_(FruitVeg) | 5 | Unknown |  | RP | 469.61 | 599.4458 |  |  |  |  | 0.18 | 0.90 (0.78-1.03) | 0.13 |
| RNC144.502.93_(FruitVeg) | 2 | PC38:6 | E | RN | 502.93 | 850.5605 (840.5319. 851.5642. 918.5478. 919.5508) |  |  |  |  | 0.15 | 0.97 (0.82-1.15) | 0.76 |
| RNC199.502.97_(FruitVeg) | 5 | Unknown | E | RN | 502.97 | 1656.1190 (791.5413. 1657.1230. 1658.1260) |  |  |  |  | 0.16 | 0.95 (0.82-1.10) | 0.50 |

^a^ Peak with same mass and isotope pattern (not shown) as the peak eluting at 310 s. indicating it to be an isomer.

*
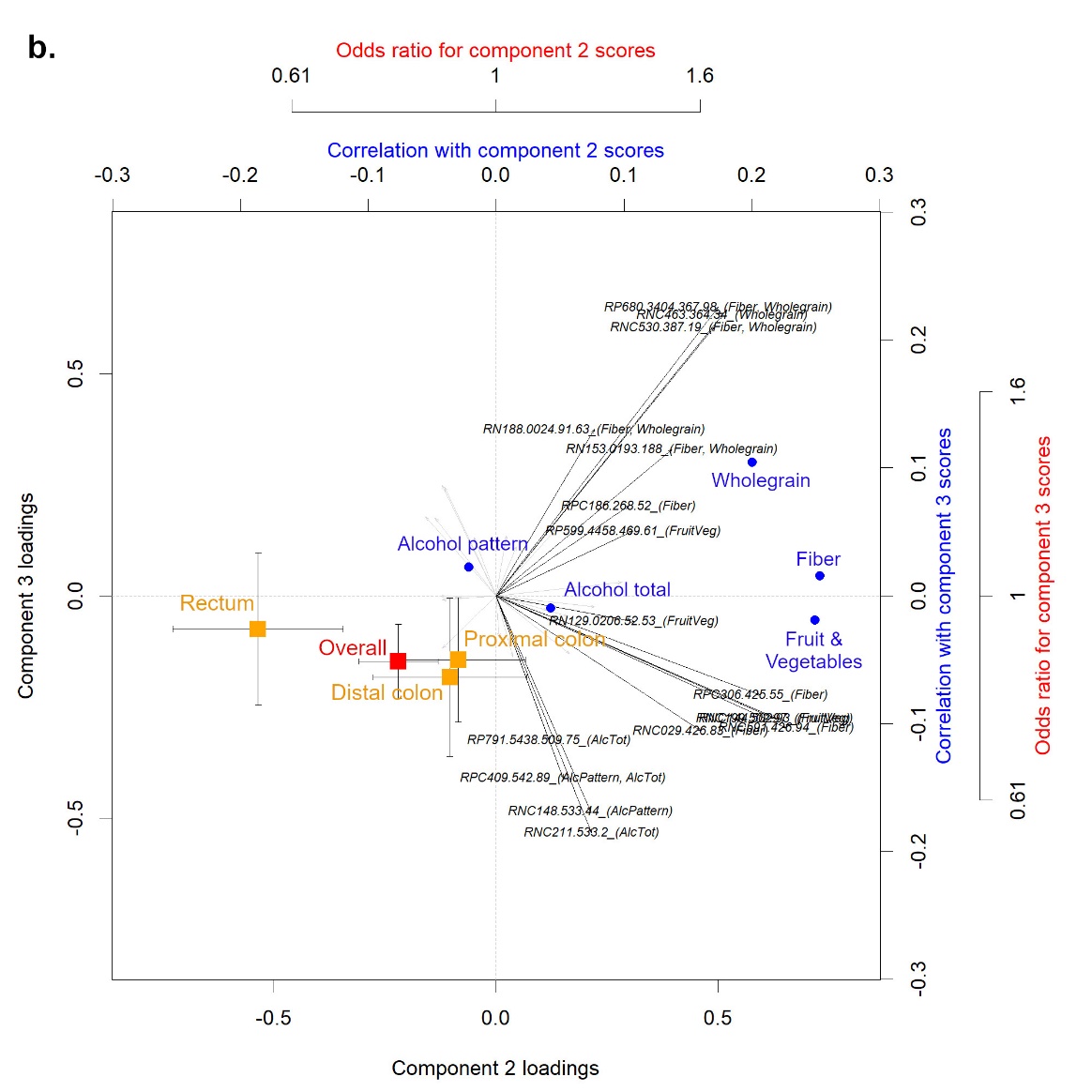
*

***
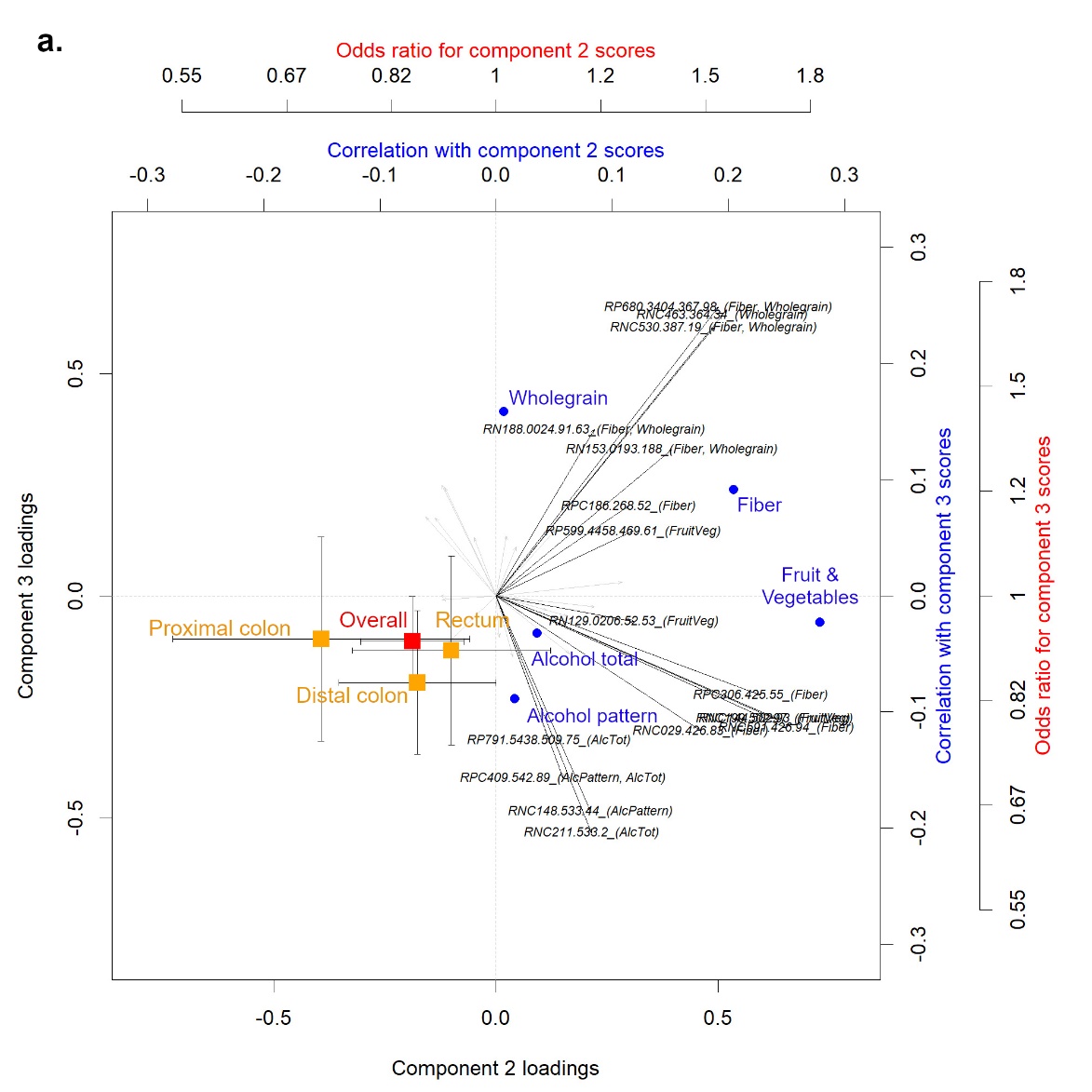
*Supplementary Figure 1.** TriPlots for **a) men** and **b) women** displaying metabolite loadings from principal component analyses (PCAs) of metabolites features selected to reflect dietary exposures (n=36).

Metabolomic feature names are reported as unique identifier (characteristics reported in Suppl Table 3; most metabolite identities are unknown) followed by the dietary pattern they reflect (in parentheses). Component scores were associated to colorectal cancer risk estimated by odds ratios (in red and orange. with whiskers denoting standard error) and to dietary exposures using partial Spearman correlation (in blue). adjusted for body mass index. smoking status. recreational physical activity. educational level. total energy intake. and alcohol intake (for association to alcohol pattern/total. alcohol was not included as a confounder).

1 Schymanski. E. L. *et al.* Identifying small molecules via high resolution mass spectrometry: communicating confidence. *Environ. Sci. Technol.* **48**. 2097-2098 (2014). <https://doi.org:10.1021/es5002105>
